# Reasons for Non-Participation in an Actigraphy Study in an Alzheimer’s Disease Center Registry

**DOI:** 10.1093/geroni/igaa057.886

**Published:** 2020-12-16

**Authors:** Alex Laffer, Hilary Hicks, Genna Losinski, Amber Watts

**Affiliations:** 1 University of Kansas, Lawrence, Kansas, United States; 2 The University of Kansas, Lawrence, Kansas, United States

## Abstract

When recruited individuals decline to participate in research, it can lead to sampling bias, increased costs, and extended duration of data collection. Understanding reasons why eligible participants decline participation may improve study enrollment rates. We aimed to understand barriers to recruitment and data collection in older adults with and without Alzheimer’s disease in the University of Kansas Alzheimer’s Disease Center Registry annual visit. We recruited Registry participants to join an observational sub-study using wrist-worn actigraphy to measure physical activity and sleep. We analyzed reasons for non-enrollment from encounters with non-participating individuals. Of 104 encounters, 37 were never recruited due to appointment cancellation, rescheduling, or no-show. Of the remaining encounters, the most common reasons for non-participation were physical limitations (N = 13), study logistics (e.g., limited supplies; N = 12), participant travel plans (N = 10), and unknown (N = 8). Other categories (N = 6) included disinterest, study partner concerns about pragmatics (e.g., fear that an individual with AD would lose the ActiGraph), problems with the study design (e.g., lack of feedback to participants), and participants’ limited availability or deferment to a later date. These findings offer insight into potential avenues to overcome barriers to participation in older adults already engaged in ongoing research through an Alzheimer’s Disease Center Registry. Researchers could benefit from adapting study procedures to correct for reasons of non-participation. For example, giving more education and reassurance to potential participants about observation and giving feedback regarding activity patterns.

